# BACTERIAL COLONIZATION IN ORTHOPEDIC SURGICAL TOURNIQUETS: A MULTICENTER STUDY

**DOI:** 10.1590/1413-785220263404e303555

**Published:** 2026-07-17

**Authors:** Eduardo Frois Temponi, Luiz Lorentz Salzmann Lamego, Eduardo Louzada da Costa, Glauco Almeida Passos, Tiago Soares Baumfeld, Leandro da Silva Marinho, Túlio Vinícius de Oliveira Campos, Raquel Bandeira da Silva, Vitor Rodrigues Miranda

**Affiliations:** 1 Pontificia Universidade Catolica de Minas Gerais, Hospital Madre Teresa, Belo Horizonte, MG, Brazil.; 2 Hospital da Unimed BH, Belo Horizonte, MG, Brazil.; 3 Hospital Evangelico de Belo Horizonte, Belo Horizonte, MG, Brazil.; 4 Hospital Felicio Rocho, Belo Horizonte, MG, Brazil.; 5 Universidade Federal de Minas Gerais, Belo Horizonte, MG, Brazil.; 6 Hospital da Unimed, Hospital da Baleia, Belo Horizonte, MG, Brazil.; 7 Universidade Federal de Minas Gerais, Hospital Risoleta Tolentino Neves, Belo Horizonte, MG, Brazil.; 8 Hospital Universitario Ciencias Medicas, Faculdade de Medicina Ciencias Medicas, Belo Horizonte, Minas Gerais, Brasil.

**Keywords:** Tourniquets, Cross Infection, Orthopedic Procedures, Bacterial Infections, Brazil, Torniquetes, Infecção Cruzada, Procedimentos Ortopédicos, Infecções Bacterianas, Brasil

## Abstract

**Objective:**

To evaluate the prevalence and microbiological profile of contamination in reusable tourniquets in Brazilian hospitals.

**Methods:**

A multicenter study conducted in six hospitals. Swabs were collected from 54 tourniquets immediately after surgical use and before disinfection, covering an estimated area of 10 cm². The samples were cultured and identified using automated methods. The microbial load was described by median and interquartile range (IQR), and comparisons between public and private hospitals were performed using Fisher's exact test and Mann-Whitney test.

**Results:**

The prevalence of contamination was 70.4% (38/54). The median overall microbial load was 101 CFU per device (IQR: 0–153), corresponding to approximately 10.1 CFU/cm². The predominant microorganisms were coagulase-negative Staphylococcus (48.1%) and Staphylococcus aureus (18.5%), with isolation of Pseudomonas aeruginosa, Bacillus sp., and Candida sp. The contamination rate was 78.6% in public hospitals and 61.5% in private hospitals (p=0.081), with no statistically significant difference in the median bacterial load between the institutions (p=0.412).

**Conclusion:**

There is a high prevalence of contamination by clinically relevant pathogens in reusable tourniquets, regardless of the type of hospital. The results indicate systemic failures in reprocessing and suggest the need for high-level disinfection protocols or the adoption of disposable sterile devices to mitigate the risk of cross-contamination. ***Level of evidence III; multicenter cross-sectional study of microbiological prevalence.***

## RESUMO

**Objetivo:**

Avaliar a prevalência e o perfil microbiológico da contaminação em torniquetes reutilizáveis em hospitais brasileiros.

**Métodos:**

Estudo multicêntrico conduzido em seis hospitais. Foram coletados swabs de 54 torniquetes imediatamente após o uso cirúrgico e antes da desinfecção, abrangendo uma área estimada de 10 cm². As amostras foram cultivadas e identificadas por métodos automatizados. A carga bacteriana foi descrita por mediana e intervalo interquartil (IIQ), e as comparações entre hospitais públicos e privados foram realizadas pelos testes exato de Fisher e de Mann-Whitney.

**Resultados:**

A prevalência de contaminação foi de 70,4% (38/54). A mediana da carga microbiana geral foi de 101 UFC por dispositivo (IIQ: 0–153), correspondendo a aproximadamente 10,1 UFC/cm². Os microrganismos predominantes foram Staphylococcus coagulase-negativo (48,1%) e Staphylococcus aureus (18,5%), com isolamento de Pseudomonas aeruginosa, Bacillus sp. e Candida sp. A taxa de contaminação foi de 78,6% nos hospitais públicos e de 61,5% nos hospitais privados (p=0,081), sem diferença estatisticamente significativa na mediana da carga bacteriana entre as instituições (p=0,412).

**Conclusão:**

Há alta prevalência de contaminação por patógenos clinicamente relevantes em torniquetes reutilizáveis, independentemente do tipo de hospital. Os resultados indicam falhas sistêmicas no reprocessamento e sugerem a necessidade de protocolos de desinfecção de alto nível ou a adoção de dispositivos estéreis descartáveis para mitigar o risco de contaminação cruzada. ***Nível de evidência III; estudo transversal multicêntrico de prevalência microbiológica.***

## INTRODUCTION

The use of tourniquets in orthopedic surgeries is a widely established practice recognized for its role in reducing intraoperative bleeding and improving the visibility of the surgical field^1–3^. However, reusable devices, with often insufficient cleaning between procedures, have been associated with high rates of microbial colonization^1,3–5^. Even without direct contact with the operative field, tourniquets maintain close contact with the skin and adjacent tissues, which may allow the transmission of pathogenic microorganisms.

International studies report contamination rates between 68% and 96% in reusable tourniquets, including the presence of *Staphylococcus aureus*, *Pseudomonas aeruginosa*, and multidrug-resistant microorganisms^2,3,5–7^. In contrast, sterile single-use devices exhibit colonization rates close to zero^3,8,9^. Ahmed et al.^4^ demonstrated that disinfection with chlorhexidine wipes can reduce the microbial load by up to 99% in tourniquets. Szymczyk et al.^5^, in turn, identified averages of up to 545 CFU/cm^2^ in reused devices, especially in emergency services.

Despite the importance of the topic, there is a lack of national data documenting the magnitude of the risk in our country^9^. Moreover, there is an absence of specific regulations from agencies such as the National Health Surveillance Agency (ANVISA), which contributes to the heterogeneity in disinfection practices adopted among different hospital institutions^10^.

In light of this scenario, the present multicenter study aims to evaluate the prevalence and microbiological profile of microbial load colonization in reusable tourniquets used in orthopedic surgeries in Brazil^11^. It also seeks to discuss the implications of these findings for healthcare-associated infection control, proposing evidence-based strategies that may underpin institutional policies and national biosafety guidelines.

## METHODOLOGY

### Design and Ethical Aspects

This was an observational, cross-sectional, and multicenter study with a quantitative approach, conducted in conducted in six Brazilian hospitals. The participating hospitals were coded for analysis and are not individually identified in the results. The study was approved by the Institutional Research Ethics Committee through the Plataforma Brasil system, under the substantiated opinion No. 7.621.206 and CAAE 89171425.0.0000.51271.

### Sample Calculation

Based on Thompson et al.^3^ and Ahmed et al.^4^, which reported contamination rates between 68% and 96%, we assumed an expected prevalence of 80% for bacterial colonization. Considering a margin of error of 10% and a confidence interval of 95%, a minimum sample of 50 tourniquets was estimated to ensure adequate descriptive power.

### Sample Collection

Fifty-four reusable tourniquets used in orthopedic surgeries (elective and emergency) between May and July 2025 were analyzed. The collection followed consecutive sampling and was conducted immediately after surgical use and before the routine institutional disinfection procedure.

A sterile swab was used for each device, rubbed in a rotational and unidirectional manner over the internal surface of the tourniquet (the area in contact with the skin). The collection protocol standardized the friction over a linear extent of 10 cm. Considering the average width of the swab tip of approximately 1 cm, the total sampled area was estimated at 10 cm^2^ for the purposes of calculating bacterial density and benchmarking (CFU/cm^2^). To ensure standardization and reliability, all collectors were previously trained according to a detailed operational protocol, which included specific instructions regarding the area to be rubbed, the contact time, and the aseptic handling of the material, minimizing inter-observer variations and potential biases related to the collection technique. The time between collection and laboratory processing was standardized, limited to a maximum of two hours, in order to ensure the microbiological viability of the samples.

### Microbiological Processing

The samples were inoculated onto 5% blood agar plates and MacConkey agar plates (Oxoid^™^), incubated at 37°C for 24 to 48 hours under aerobic atmosphere. The identification of microorganisms was performed using automated methods (VITEK® 2 Compact, bioMérieux or MALDI-TOF, Bruker®). The quantification of bacterial load was conducted through direct counting of Colony Forming Units (CFU). The microbiologists responsible for reading were blinded to the type of hospital from which the samples originated.

### Data Analysis

The data were analyzed using R software (version 4.3.1). The normality of the quantitative variables was assessed using the Shapiro-Wilk test. Due to the non-parametric distribution of the data, the bacterial load (CFU) was described by median and interquartile range (IQR: Q1-Q3). The categorical variables were described in absolute and relative frequencies.

To compare proportions between public and private hospitals, Fisher's exact test was used. To compare bacterial loads (non-parametric continuous variables), the Mann-Whitney test was employed. A significance level of 5% (p<0.05) was adopted for all analyses.

## RESULTS

Out of the 54 analyzed tourniquets, 38 exhibited microbial growth, corresponding to a prevalence of 70.4% (95% CI: 57.4% – 81.1%). There was no microbial growth in 16 tourniquets (29.6%).

### Microbiological Profile

The frequency of isolated microorganisms is detailed in Table 1 (Frequency of isolated microorganisms). The microorganism most frequently isolated was coagulase-negative Staphylococcus, present in 26 samples (48.1%). Pathogens of clinical relevance were identified, including *Staphylococcus aureus* (18.5%), *Bacillus sp*. (14.8%) and *Pseudomonas aeruginosa* (9.2%). In addition to bacteria, yeasts of the genus *Candida spp.* were isolated in 2 samples (3.7%). In two tourniquets, co-colonization by more than one microorganism was detected simultaneously (Table 1).

**Table 1:** Frequency of isolated microorganisms.

Isolated Microorganism	n (isolates)	Frequency (%)*
Coagulase-negative Staphylococcus	26	48.1%
Staphylococcus aureus	10	18.5%
Bacillus sp.	8	14.8%
Pseudomonas aeruginosa	5	9.2%
Candida spp.	2	3.7%

### Bacterial Load

Because bacterial load was not normally distributed of microbial load (Shapiro-Wilk p<0.05). The estimated overall median was 101 CFU per device (Interquartile Range [IQR]: 0 – 153). When adjusted for the sampled surface area (10 cm^2^), the median density was 10.1 CFU/cm^2^.

### Comparison between Institutions

**Figure 1:**
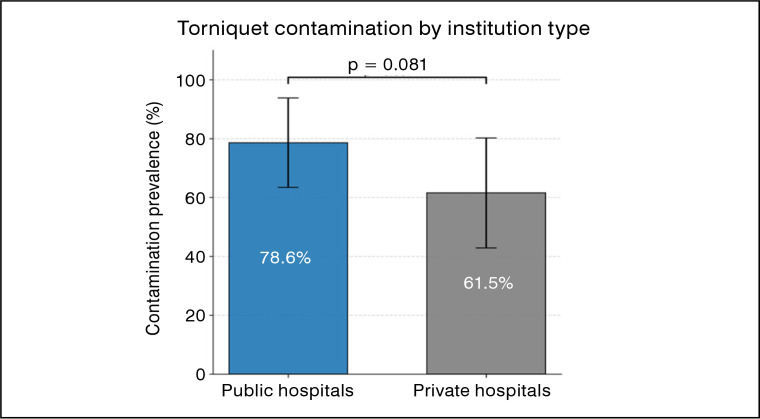
Prevalence of contamination by type of hospital.

**Figure 2:**
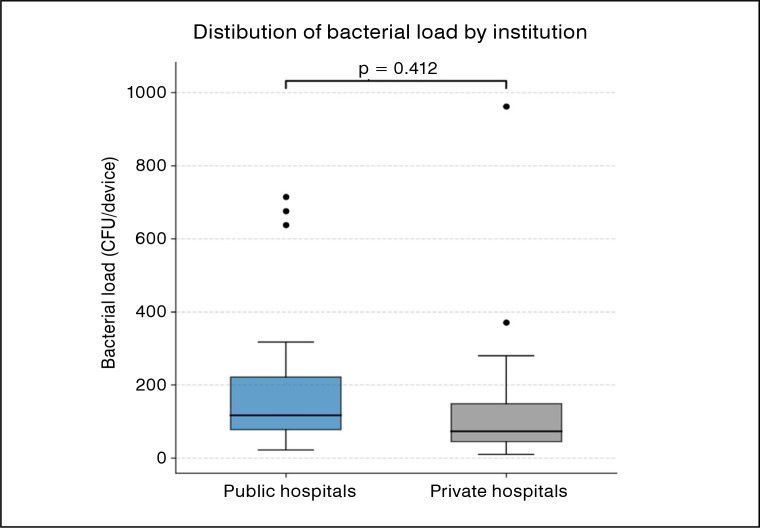
Distribution of bacterial load (CFU) by type of hospital.

Of the 28 tourniquets from public hospitals, 22 exhibited contamination (78.6%). Among the 26 tourniquets from private hospitals, 16 were contaminated (61.5%). Despite the absolute percentage difference, this variation did not reach statistical significance (p=0.081; Fisher's exact test). (Figures 1 and 2)

Regarding microbial load, devices from public hospitals had a median of 117 CFU (IQR: 85 – 184), while those from private hospitals had a median of 73 CFU (IQR: 0 – 148). The comparison of bacterial load distributions between the two groups also did not demonstrate a statistically significant difference (p=0.412; Mann-Whitney test).

## DISCUSSION

The results of this multicenter study reveal a prevalence of microbial colonization of 70.4% in reusable orthopedic tourniquets, corroborating international data that report contamination rates ranging from 68% to 96% in similar devices^1,3–5^. The detection of a global median of 101 CFU per device (estimated at ~10.1 CFU/cm^2^), immediately after intraoperative use and before reprocessing, evidences a substantial biological load that challenges the current disinfection protocols. In contrast, studies demonstrate that sterile single-use devices or those subjected to sterilization protocols exhibit colonization rates close to zero, highlighting the role of reusable tourniquets as frequently overlooked high-touch contact surfaces^3^. The identified microbiological profile exceeds the harmless commensal microbiota, with a high prevalence of *coagulase-negative Staphylococcus* (48.1%) and *Staphylococcus aureus* (18.5%), aligning with recent findings by Szymczyk et al.^5^. This scenario represents a high clinical risk, as these agents are the main causes of periprosthetic infections, and the literature warns of the potential for these devices to act as reservoirs for multidrug-resistant organisms, perpetuating cycles of hospital infection^6^. Additionally, the presence of *Pseudomonas aeruginosa* (9.2%) and *Bacillus* sp. (14.8%) suggests failures in the biosafety barrier and environmental persistence of biofilm-forming organisms, which are difficult to eradicate through simple manual cleaning, while the isolation of *Candida spp.* (3.7%) reinforces the complexity of contamination favored by moisture retained in the tissues of the cuffs.

The association between the use of tourniquets and the increased risk of Surgical Site Infection (SSI) is well documented, with meta-analyses indicating a high risk in total knee arthroplasties^1,8,12^. Although factors such as tissue hypoxia have historically been blamed, recent evidence demonstrates that the tourniquet also affects the local tissue concentration of prophylactic antibiotics, compromising the effectiveness of perioperative prophylaxis^2^. In addition, the physical presence of a source rich in viable pathogens in the immediate vicinity of the surgical field constitutes a critical modifiable risk vector. We recognize as a limitation of this cross-sectional study the absence of "pre-use" immediate collection, which prevents the unequivocal distinction of whether the recovered microbial load originates from the skin flora of the current patient or represents residual contamination from previous procedures. However, from the perspective of biosafety, this distinction becomes secondary in light of the risk of cross-contamination for the next patient. The documented bacterial load (median of 117 CFU in public hospitals and 73 CFU in private ones) represents the biological challenge that the subsequent cleaning process must eliminate. Ahmed et al.^4^ demonstrated that, although cleaning with chlorhexidine may reduce the load by 99%, the effectiveness in routine practice is inconsistent and operator-dependent. If reprocessing is ineffective, the identified pathogens will persist, transforming the tourniquet into a passive vector for the subsequent surgical case. The comparison between public and private hospitals did not demonstrate a statistically significant difference in contamination rates (p=0.081) or in the median bacterial load (p=0.412), clinically suggesting that the contamination of tourniquets is a systemic problem in orthopedic practice, regardless of management model or resource availability. The persistence of contamination above 60% in both groups indicates that current protocols based on manual cleaning are insufficient. The adoption of alternative materials, such as silicone tourniquets, has demonstrated a reduction in contamination rates compared to traditional fabric ones, presenting itself as a viable alternative to reduce microorganism adhesion^10^. Furthermore, systematic reviews reinforce that the concern for the safe use of the tourniquet should be universal, encompassing everything from pediatrics to complex reconstructive surgeries^7^. Although the acquisition of disposable sterile tourniquets represents a higher initial cost, the economic analysis should consider the burden of treating complications, as the clinical impact of failures in arthroplasties justifies preventive investments^13^. The aggregated cost of treating a single deep infection exponentially exceeds the investment in adopting single-use devices, in addition to mitigating the spread of resistant microorganisms in the hospital environment^9,11^. For institutions where the transition to disposables is not immediate, it is recommended to validate high-level disinfection protocols, the mandatory use of waterproof sterile protection (*stockinette*) under the tourniquet, and periodic monitoring of microbial load.

Despite the relevance of the findings for national biosafety, this study presents limitations that must be considered in the interpretation of the results. The main restriction lies in the cross-sectional design without an immediate "pre-use" baseline collection, which prevents the unequivocal distinction between contamination arising from the skin microbiota of the current patient and the residual load resulting from failures in the reprocessing of previous surgeries. Although this limitation restricts the precise definition of the origin of the inoculum, it does not invalidate the finding of the risk of cross-contamination, as the detected biological load represents the real challenge to be eliminated before the next use. Additionally, the swab sampling technique, although standardized, may underestimate the total microbial load, especially those deeply adhered to the fabric of the cuff or organized in biofilms, which would be better recovered by sonication methods, logistically unfeasible in the proposed multicenter design.

Another important limitation was the lack of antimicrobial sensitivity testing (antibiogram), which hindered the characterization of the resistance profile of the isolates, such as the prevalence of *S. aureus* resistant to methicillin (MRSA). The absence of negative field controls (swabs exposed to the environment without contact with the device) also prevents the exact quantification of background environmental contamination, although the rigor in the aseptic technique aimed to minimize this bias. Moreover, clinical covariate variables, such as the duration of surgery, type of antiseptic skin preparation, or the use of protective meshes (*stockinettes*) under the tourniquet, factors that could influence the final colonization density, were not collected. Finally, as the study focused on the colonization of the device and did not follow patients longitudinally, it is not possible to establish a direct causal correlation between the contamination of the tourniquets and the rates of surgical site infection (SSI) in the participating institutions, leaving this inference based on biological plausibility and comparative literature.

## CONCLUSION

This multicenter study highlights a high prevalence of microbial colonization (70.4%) in reusable orthopedic tourniquets in Brazil, with the identification of microorganisms of high clinical relevance, including *Staphylococcus aureus*, *Pseudomonas aeruginosa*, and *Candida* spp. The absence of a statistically significant difference in contamination rates and microbial load between public and private hospitals indicates that the insufficiency of reprocessing protocols is a systemic and cross-sectional challenge in national orthopedic practice, not limited to scenarios with resource constraints. It is concluded that reusable tourniquets, when subjected only to conventional cleaning, act as potential reservoirs of pathogens in the surgical environment. In light of these findings, it is recommended to review institutional biosafety guidelines, prioritizing the adoption of sterile disposable tourniquets, especially in implant surgeries, or, in the impossibility of this transition, the rigorous implementation of high-level disinfection associated with the mandatory use of waterproof sterile barriers.

## Data Availability

The underlying contents of the research text are contained in the manuscript.
